# Period and prejudice: Cultural differences in menstrual health between South Asian and White British women

**DOI:** 10.1177/17455057261478339

**Published:** 2026-09-28

**Authors:** Michèle D. Birtel, Lucia Volpi, Aisha Mirza, Laura M. König

**Affiliations:** 1 Institute for Lifecourse Development, 411470 University of Greenwich, London, UK; 2 Faculty of Psychology, University of Vienna, Vienna, Austria; 3 4586 Mersey Care NHS Foundation Trust, Prescot, UK

**Keywords:** menstrual health, stigma, literacy, culture, microaggressions

## Abstract

**Background:**

Attitudes towards menstruation are shaped by cultural and social norms and can result in misperceptions, impacting women’s health. Yet, research on cultural differences in menstrual health remains limited.

**Objectives:**

This study aimed to examine whether South Asian and White British women living in the UK differ in menstrual health, with a focus on menstrual experiences, menstrual literacy, stigma-related factors, and ethnic microaggressions in the healthcare context.

**Design:**

White British (*n* = 153) and South Asian (*n* = 153) menstruating women living in the UK participated in a preregistered cross-sectional online survey.

**Methods:**

Menstrual experiences were assessed via menstrual distress, symptom management, and hygiene behaviors. Menstrual stigma was measured through affective internalized stigma, internalized stigma of seeking help, secrecy, and avoidance behaviors.

**Results:**

In addition to differences in menstrual experiences (menstrual pain, symptom management, hygiene behaviors), South Asian women reported lower menstrual literacy than White British women. South Asians also reported greater secrecy as well as stronger avoidance behaviors related to discussing menstruation and engaging in daily activities while menstruating, but also more positive emotions towards menstruation. Additionally, they experienced more ethnic microaggressions in healthcare. Secrecy and microaggressions mediated the effect of cultural group on self-stigma of seeking help.

**Conclusion:**

Our findings suggest cultural differences in menstrual health, emphasizing the need for inclusive health education and stigma-reduction interventions. Addressing menstrual literacy gaps and tackling stigma in families and microaggressions in healthcare settings may improve support for South Asian women in the UK.

## Introduction

Menstrual health is a key aspect of public health, and the menstrual cycle is a key indicator of women’s health.^1^ In line with the World Health Organization’s definition of health, menstrual health is “a state of complete physical, mental, and social well-being and not merely the absence of disease or infirmity, in relation to the menstrual cycle”^2^ (p.23). Improving menstrual health is essential for global health and aligns with the UN Sustainable Development Goals on health (SDG 3) and gender equality (SDG 5). Menstrual health impacts physical health (e.g., reproductive tract infections), mental health (e.g., depression), and social wellbeing (e.g., attending school or work).

Menstrual stigma, rooted in broader social, economic, and structural inequalities, perpetuates and exacerbates gender inequality.^3^ The impacts of menstrual stigma on mental and physical health, as well as social wellbeing, are particularly harmful for marginalized groups such as people from ethnic minority backgrounds.^4,5^ Education is inadequate, and marginalized group members can be reluctant to seek help for menstrual pain or other symptoms due to stigma from healthcare professionals.^6,7^ However, menstrual health remains understudied in cross-cultural contexts. Most studies on menstrual stigma examine cultures in isolation or across countries, overlooking the experiences of minority groups within dominant cultural settings. Little is known about how menstrual experiences, literacy, and stigma operate where cultural norms and marginalization intersect, limiting understanding of related health inequalities. The aim of this study therefore was to examine whether South Asian and White British women living in the UK differ in menstrual health, with a focus on 1) menstrual experiences, 2) menstrual literacy, 3) stigma-related factors, and 4) ethnic microaggressions in the healthcare context.

### Menstrual health in different cultures

#### Menstrual experiences

Experiences of *menstrual symptoms* are common, with 85% of women reporting pain and 77% reporting psychological complaints.^8^ Yet, findings on cross-cultural differences are inconsistent: Some studies find higher levels of dysmenorrhea or premenstrual and menstrual symptoms among Western populations (e.g., Australians, Caucasians, Americans) compared to Oriental, Indian or Afro-Caribbean populations,^9–11^ while others report the opposite pattern.^12^ The substantial heterogeneity in study populations (e.g., age range, presence of menstrual disorders) and methodological approaches limits comparability across studies and conclusions that can be drawn, requiring further research. For this reason, rather than testing hypotheses, we explored differences in *menstrual distress* between White British and South Asian women.

The findings on menstrual distress mirror research findings on ethnic differences in pain sensitivity more generally, including greater pain sensitivity among minority groups,^13,14^ with South Asians in the UK reporting more intense, unpleasant pain and lower thresholds than White British women.^15^ This supports a biopsychosocial account and raises the question of whether differences are biological or shaped by social and psychological factors. For example, menstrual pain may be more readily normalized in some Eastern contexts,^9^ whereas Western discourses simultaneously medicalize and trivialize menstruation,^1,16–18^ framing changes as symptoms and blood as pollution, yet as mundane or ‘not real’. Regarding *menstrual symptom management*, use of pain medication and psychological therapy as well as social support might thus be greater among White British women, with mental health stigma, but also cultural views on suffering, limiting therapy use and perceived social support among South Asian women.^19,20^ Due to the lack of literature for the other types of menstrual symptom management, we explored differences for these.

Cultures also differ in their *menstrual hygiene behaviors*, with White people being more likely to use unscented tampons and menstrual cups than Black people, whereas Black people are more likely to use douches and wipes than White people.^21,22^ Systematic and avoidable differences in menstrual health lead to negative physical (e.g. reproductive tract infections) and emotional (e.g. anxiety, distress) outcomes.^23^ Given the lack of research with South Asians, rather than testing hypotheses, we explored differences in menstrual hygiene behaviors between White British and South Asian women.

#### Menstrual health literacy

Menstrual health literacy can be seen as a subcomponent of health literacy, and understood as the set of cognitive and social abilities that enable individuals to access, interpret, and utilize information concerning menstruation, with the aim of promoting good menstrual health and well-being.^24^ Knowledge about menstruation remains limited in both Western and Eastern cultures, but with distinct patterns. In Eastern cultures, adolescent girls are often uninformed or misinformed before menarche, typically learning from their mothers. In Western cultures, formal education often provides higher initial knowledge and preparedness, yet discomfort in discussing menstruation continues to hinder comprehensive understanding.^9,25–29^ We predicted that White British women would report greater menstrual health literacy, in particular regarding the causes of menstruation, than South Asian women.^26^ We explored differences for the other menstrual literacy items, as South Asian women in the UK in theory have similar access to education as White British women, yet educational inequalities in the UK persist.^30^

#### Menstrual stigma

Menstruation is a universal physiological process, yet misconceptions and negative attitudes toward it are widespread, harming the rights and quality of life of those who menstruate.^7,31^ Public stigma arises through social and cultural interactions that mark individuals as different and devalued, leading to discrimination.^32^ Menstrual stigma fits all three of Goffman’s (1963) categories of stigma^16^: it frames the body as unclean, the character as flawed when periods are visible, and the group identity as inferior by contrasting menstruating women with the normative male body. Stigma from the general public can be internalized by the stigmatized individual. A core aspect of internalized menstrual stigma is concealment.^33^ Terror management theory suggests that menstruation symbolizes human vulnerability, prompting existential anxiety and the urge to hide it.^34^ Objectification theory further explains that women may internalize the male gaze and engage in self-objectification, motivating concealment of menstruation.^16,35^ Stigma is reinforced by taboos and media messages that stress secrecy to avoid embarrassment. Women who view menstruation as shameful are more likely to change behavior to withdraw from public life.^36^

Menstrual stigma exacerbates gender and health inequalities, and is embedded in historical, cultural, and religious beliefs.^7,37,38^ In many Eastern cultures, menstruation is viewed positively as a natural process, yet simultaneously regarded as impure. In Western cultures, patriarchal perspectives dominate, framing female bodies as inferior and menstruation as dirty.^9,16,26,33,38,39^ Stigma manifestation takes overt and covert forms depending on cultural setting. In Eastern cultures, stigma tends to be visible, expressed through formal physical separation or restrictions on activities such as worship or household. Across South Asian contexts, menstruation is often framed as a temporary state of impurity in which women are considered ‘ritually unclean’, and therefore forbidden to pray or engage in sexual relations until after they are deemed pure, either after the menstruation has passed or after they have engaged in specific cleansing practices at the end of the menstruation. While there is religious heterogeneity and religion can play a part, South Asians tend to collectively hold such beliefs.^40,41^ In Western cultures, stigma is more hidden, reflected in expectations to conceal menstruation, use euphemisms, and maintain silence, resulting in the topic rarely being discussed openly.^7,16,38^ Due to the physical separation in South Asian cultures, Western cultures may have a higher tendency to rely on social support to manage their menstrual symptoms than Eastern cultures. Also, help-seeking patterns in the context of menstruation somewhat differ between cultures. In Eastern cultures, women often experience delayed diagnosis and under-treatment of menstrual disorders, compounded by discomfort in consulting male doctors. In Western cultures, delays and under-treatment also occur, though the discomfort with male practitioners is less emphasized.^28^ We therefore predicted that South Asian women would report greater stigma towards menstruation than White British, specifically in terms of affective internalized stigma, internalized stigma of seeking help, secrecy, and avoidance behaviors relating to men, male health professionals, prayers, and sexual intimacy. Given that menstruation is viewed more positively in South Asian cultures, we also measured positive emotions associated with menstruation, to identify potentially distinct affective stigma profiles across cultural groups, rather than assuming stigma is exclusively negative.

#### Ethnic microaggressions in healthcare

In the UK, South Asian women not only navigate cultural norms around menstruation but also face the challenges of minority status and discrimination in healthcare. Research findings with South Asian women in their countries of origin may not necessarily generalize to South Asian women living in a Western dominant society like the UK.^19^ In such settings, South Asian women are both an ethnic minority and embedded in a social environment with different cultural norms. Consequently, they may face public stigma not only related to menstruation but also linked to their minority status.^42^ For example, South Asian patients reported being discriminated against by primary care services, and concerns about a lack of cultural awareness.^43^ Perceived discrimination has been shown to negatively impact both mental and physical health.^44^ Microaggressions are a more subtle, indirect but especially impactful form of prejudice that manifests in brief, everyday interactions as microinsults, microinvalidation, or microassault. Significant harm to physical and mental health is caused due to their ambiguity and repetition.^45^ We predicted that South Asian women would experience greater ethnic microaggressions in healthcare when seeking help for menstrual symptoms than White British.

### The present study

Taken together, research shows that menstrual health is shaped by cultural norms and social structures, with important consequences for wellbeing and help-seeking. While studies within different cultures have highlighted differences in menstrual experiences, literacy, and stigma, research directly comparing menstrual experiences, literacy and stigma between Eastern and Western cultures is limited, with mixed results.^9–12^ In addition, little is known about how these factors manifest across cultural groups within the same national context,^12,22^ considering the group status (majority vs. minority) and discrimination associated with such status. The current study addresses this gap by directly comparing South Asian and White British women in the UK with regard to menstrual health. Specifically, we 1) compare White British with South Asian women living in the UK as a minority group in a dominant Western society, 2) examine cultural differences in menstrual health, focusing on menstrual experiences, literacy, stigma-related factors, and ethnic microaggressions in healthcare settings, and 3) provide first insights into the process of internalized stigma.

We assessed all three dimensions (i.e., physical, mental, social) of menstrual health^2^ during the menstruation phase. This phase was selected because of its relevance to both internalized and public stigma: concerning the former, menstrual blood is the most concealed menstrual mark by people who menstruate^46,47^; as for the latter, menstruation (i.e., bleeding phase) is the most subjected to cultural prescriptions (e.g., ^43^). Menstrual distress is defined as the interference with quality of life,^48^ recreational or work activities, and social relationships resulting from menstrual cycle symptoms.^49^ We preregistered^50^ and tested the following hypotheses for the respective research questions:

#### Menstrual experiences

*RQ1:* Do South Asian and White British women differ in their self-reported menstrual experiences?

Hypothesis 1
South Asians and White British differ in the types of menstrual symptom management. White British (vs. South Asians) report the use of social support (family, partner and friends); pain medication; and psychological therapy more often.

#### Menstrual literacy

*RQ2:* Do South Asian and White British women differ in their menstrual literacy?

Hypothesis 2
South Asians and White British differ in menstrual literacy: White British are more likely to attribute the cause of menstruation to a physiological cause than South Asians.

#### Menstrual stigma

*RQ3:* Do South Asian and White British women differ in menstrual stigma?

Hypothesis 3
South Asians and White British differ in internalized menstrual stigma: South Asians (vs. White British) report greater affective internalized stigma, greater internalized stigma of seeking help, greater secrecy, greater avoidance behaviors relating to men, male health professionals, prayers, and sexual intimacy.

#### Ethnic microaggressions in healthcare

*RQ4:* Do South Asian and White British women differ in their experienced ethnic microaggressions in healthcare?

Hypothesis 4
South Asians report greater experienced ethnic microaggressions in the healthcare context than White British.

#### Mediations

We also tested hypotheses relating to the process of internalized stigma (see Supplementary Material Section B: Mediation Models Figures S1-S4). Affective internalized stigma and secrecy mediate the association between cultural group and: avoidance behaviors (men, male health professionals, prayers, sexual intimacy) (*Hypothesis 5*), social support (family, partner, friends) as symptom management (*Hypothesis 6a*), and pain medication as well as psychological therapy as symptom management (*Hypothesis 6b).*

Hypothesis 7
Internalized stigma of seeking help mediates the association between experienced ethnic microaggressions in healthcare and menstrual symptom distress.

## Method

Preregistration, material, and data are available on the Open Science Framework.^50^ The research received ethical approval from the local institutional ethics committee (Ref: 23.5.5.10, date: 08/07/24). Informed consent to participate and for publication was obtained from participants in writing. The STROBE cross-sectional reporting guidelines were followed.^51^

### Participants

Participants were 153 South Asian and 153 White British individuals living in the UK (*N*_total_ = 306) who identified as cis women, had menstruated in the past 12 months and were aged between 18 and 50 years. The sample was restricted to cisgender women to limit heterogeneity related to intersecting forms of stigma (e.g., related to gender identity), which were beyond the scope of the present study. They were recruited from the general UK population via an online survey on Qualtrics (duration: 30 minutes) which was distributed on Prolific. Informed consent was taken in writing from the participants prior to the beginning of the survey. For further demographics see Tables 1 and 2. Our target sample size of 300 participants was determined by the available funding for Prolific (3 GBP per participant). A power analysis using G*Power^52^ for a one-way MANOVA with two groups and 13 dependent variables indicated that a total sample size of approximately 298 participants would be required to detect a medium effect size (*f*^2^(V) = .0625) with 80% power at an alpha level of .05. A Monte Carlo power analysis for parallel mediation models (with one predictor, two mediators, and one outcome) indicated that a sample size of approximately 226 participants was required to detect medium effect sizes with 80% power at an alpha level of .05.^53^

**Table 1.** Sociodemographic characteristics of participants (frequencies).

​	White British *n* = 153	South Asian *n* = 153
**Country of birth**	​	​
UK	148	110
Europe	4	2
South Asia	0	41
Singapore	1	0
**Country of birth of parents**	​	​
UK	146	14
Europe	4	1
South Asia	0	118
Africa	0	4
Mixed (UK, Europe, Asia, Africa)	3	16
**Length of residence in the UK**	​	​
Since birth	143	107
Not since birth	10	46
Percentage of lifetime spent in the UK	*M* = 55% (*SD* = 31.61)	*M* = 35.07% (*SD* = 27.80)
**Education**	​	​
Less than upper secondary education (less than GCSE or equivalent)	3	0
Upper secondary education (GCSE or equivalent)	21	20
Post-secondary non-tertiary degree	27	19
Postgraduate certificate or equivalent	7	1
Bachelor’s degree	67	65
Master’s degree	25	42
Doctoral degree	3	4

**Table 2.** Means (standard deviations), significance tests and effect sizes for demographics, group identification, menstrual health literacy and ethnic microaggressions in healthcare as a function of cultural group.

Measure	Range	Cultural group		*t* (df)	*p*	Cohen’s *d*
		White British	South Asian			
Age	18-50	34.54 (7.69)	29.20 (8.01)	5.95 (304)	<.001	.680
Education	1-7	4.31 (1.44)	4.68 (1.41)	-2.21 (302)	.028	-.253
Subjective socioeconomic status	0-10	5.08 (1.40)	5.14 (1.53)	-0.39 (304)	.697	-.044
Group identification – British identity	1-5	4.00 (0.80)	3.57 (0.77)	4.75 (304)	<.001	.543
Group identification – Asian identity	1-5	1.67 (0.61)	3.82 (0.79)	-26.74 (304)	<.001	-3.057
Menstrual health literacy	0-5	4.42 (0.71)	4.17 (0.81)	2.92 (304)	.004	.334
Ethnic microaggressions in healthcare	1-6	1.14 (0.35)	1.47 (0.65)	-5.52 (231.09)	<.001	-.631

*Note.* Sample size for all measures *N* = 306 (153 White British, 153 South Asians).

### Procedure

Participants received information about the study prior to beginning the survey and provided informed consent to take part. Of the 309 participants that completed the consent form, three indicated they did not menstruate and were screened out before participation. All 306 participants passed the attention check. Upon completion of the survey, they were debriefed and thanked for their participation.

### Measures

The multidimensionality of menstrual health was assessed as follows:

1) Physical: The physicality dimension of menstrual health was measured by assessing menstrual hygiene behaviours (i.e., type of menstrual material used), symptom management strategies (e.g., contraceptives, diet), physical menstrual symptom distress (i.e., pain, gastrointestinal and physiological).
2) Mental: The mental dimension of menstrual health was measured by assessing the attitudes towards menstruation (i.e., internalised affective stigma and secrecy) and the psychological and cognitive symptom distress.
3) Social: The social dimension of menstrual health was measured by assessing inter-relational aspects of menstruation (i.e., help seeking due to menstrual issues, microaggressions in the healthcare context) as well as social aspects related to the management of menstruation (i.e., talking to men, talking to family members, praying), avoidance behaviours, and menstrual literacy as a proxy of menstrual education.

#### Menstrual experiences

##### Frequency and distress of menstrual symptoms

The *Menstrual Distress Questionnaire (MEDI-Q)*^49^ was used to assess the frequency and distress of menstrual symptoms during menstruation. We used the subscales relating to the menstrual phase only. The frequency subscale indicated the presence of the symptoms during the days of menstruation, and was rated on a 3-point Likert scale (0 = never, 1 = symptom occurred during less than half of the menstrual period in the last 12 months, 2 = symptom occurred during at least half of the menstrual periods in the last 12 months). The menstruation distress subscale indicated the level of distress associated with the symptoms during menstruation, and was rated on a 4-point Likert scale (0 = no distress, 1= slight distress, 2 = moderate distress, 3 = severe distress). An overall average frequency score was calculated (Cronbach’s alpha α_White_ = .92, α_Asian_ = .91), and the distress ratings for the symptom clusters were averaged into separate scores for pain (Cronbach’s alpha α_White_ = .68, α_Asian_ = .60), gastrointestinal (Cronbach’s alpha α_White_ = .73, α_Asian_ = .81), discomfort (Cronbach’s alpha α_White_ = .72, α_Asian_ = .72), psychological and cognitive (Cronbach’s alpha α_White_ = .89, α_Asian_ = .86), and other physiological (Cronbach’s alpha α_White_ = .66, α_Asian_ = .90). The low Cronbach’s alpha values for the pain cluster might be related to higher missingness in this cluster (i.e., cluster level missingness: *n*_White_ = 421/918, 45.9%; *n*_Asian_ = 426/918, 46.4%). Additionally, the heterogeneity of pain items (e.g., abdominal pain, headaches, pain during sex) may have contributed to the comparatively low internal consistency observed for this cluster.

##### Management of menstrual symptoms

Participants rated the likelihood of using 13 ways to manage their menstrual symptoms (contraceptive, pain medication, antidepressants, exercise, heat, massage, diet, psychological therapy, social support from family/partner/friends, staying at home, calling in sick) on a 5-point Likert scale (1 = not at all, 5 = very much), developed from the literature.

##### Menstrual hygiene behaviors

Participants were asked how frequently they have used each of eight products in the past year (tampons, sanitary napkins/pads, menstrual cups, douches, feminine sprays, feminine powders, feminine wipes, other) on a 7-point Likert scale (1 = never, 2 = less than once a month, 3 = 1–3 times a month, 4 = during my period, 5 = 1–5 times a week, 6 = 6 or more times per week, 7 = more than once per day) (Cronbach’s alpha α_White_ = .74, α_Asian_ = .75).^22^

#### Menstrual health literacy

Knowledge about menstruation was assessed using five multiple-choice items adapted from Hennegan et al.,^54^ Siddique et al.,^55^ and Uzma et al.,^56^ designed to capture participants’ basic knowledge about menstruation (see Supplementary Material, Section C: Scales). One question assessed beliefs about the cause of menstruation (physiological, psychological, God given, result of sin, due to some disease). Additional items asked from which organ the menstrual bleeding occurs, whether there are any toxins in menstrual blood, whether periods are under the influence of hormones, and whether there are certain days when a woman is more likely to become pregnant during the menstrual cycle. We computed frequencies of correct answers.

#### Menstrual stigma

Menstrual stigma was measured through internalized affective stigma, internalized stigma of seeking help, secrecy, and avoidance behaviors.

##### Affective internalized menstrual stigma

Affective self-stigma was assessed using the 11 clusters of emotions from the Menstrual Self-Evaluation Scale.^57^ Participants rated the extent to which they feel various emotions when thinking of their menstrual period on a 5-point Likert scale (1 = not at all, 5 = extremely) on three subscales: loathing emotions (four 3-item clusters, e.g., disgust-distaste-revulsion, angry-irritated-annoyed; Cronbach’s alpha α_White_ = .82, α_Asian_ = .86), self-conscious emotions (four 3-item clusters, e.g., embarrassed-self-conscious-blushing, ashamed-humiliated-disgraced; Cronbach’s alpha α_White_ = .87, α_Asian_ = .88), and positive emotions (three 3-item clusters, e.g., glad-happy-joyful, proud-satisfied-honorable; Cronbach’s alpha α_White_ = .78, α_Asian_ = .78). Mean scores were calculated for each subscale and for the full scale (Cronbach’s alpha α_White_ = .80, α_Asian_ = .81).^58^

##### Internalized stigma of seeking help for menstrual problems

Internalized stigma of seeking help was measured using 10 items adapted to menstrual stigma from the Self-Stigma of Seeking Help Scale (SSOSH; ^52^). Participants indicated the extent to which each statement described how they might react in the situation, using a 5-point Likert scale (1 = strongly disagree, 5 = strongly agree). The items captured self-evaluative concerns and potential threats to self-worth associated with help-seeking. Example items include: “I would feel inadequate if I sought professional help for menstrual problems” and “I would feel worse about myself if I sought professional help for menstrual problems.” Items were recoded so that a higher mean score indicated greater internalized stigma associated with seeking professional support for menstrual concerns (Cronbach’s alpha α_White_ = .83, α_Asian_ = .88).

##### Secrecy

Secrecy around menstruation was measured using 12 items from the Beliefs About and Attitudes Toward Menstruation (BATM) scale.^59^ Participants rated their agreement on a 5-point Likert scale (1 = strongly disagree, 5 = strongly agree). Sample items include: “We women must hide anything that shows that we are having our periods” and “It is uncomfortable for us women to talk about our periods”. Items were recoded so that a higher mean score indicated stronger endorsement of secrecy toward menstruation (Cronbach’s alpha α_White_ = .81, α_Asian_ = .87).

##### Avoidance behaviors

Participants rated the extent to which 11 activities should be avoided during menstruation (e.g., “talking to men about my period”, “engaging in sexual intimacy”, “exercise”, “engaging in prayers”), using a 5-point Likert scale (1 = should not be avoided at all, 5 = should very much be avoided) (adapted from^29^).

#### Ethnic microaggressions in healthcare

Experienced ethnic microaggressions in healthcare were assessed using 16 items adapted from the Daily Life Experiences (DLE) scale,^60^ adapted to reflect experiences within healthcare settings. Participants indicated how often each experience had occurred in the past year when interacting with a healthcare professional because of their ethnic background. Example items were “Being ignored, overlooked, or not given treatment,” “Being insulted, called a name or harassed,” and “Not being taken seriously.” Responses were recorded on a 6-point Likert scale (0 = never, 1 = once, 2 = a few times, 3 = about once a month, 4 = a few times a month, 5 = once a week or more). A higher mean score indicated more frequent experiences of ethnic microaggressions in healthcare contexts (Cronbach’s alpha α_White_ = .92, α_Asian_ = .94).

#### Demographics

Participants reported their sex assigned at birth, gender identity, age, whether they had menstruated in the last 12 months, sexual orientation, ethnicity, country of birth, their parents’ countries of birth, length of residence in the UK, and highest level of education.

Subjective socioeconomic status was assessed using the MacArthur Scale of Subjective Social Status.^61^ Participants were presented with an image of a ladder representing where people stand in society in the United Kingdom and were asked to indicate the rung that best reflected their perceived standing at this time in their life, compared to other people in the UK. The scale ranged from 0 (at the bottom in the UK) to 10 (at the top in the UK).

Group identification was measured separately for South Asian and White British identity using three items each (adapted from^62^): “I feel British/South Asian,” “I feel a bond with British/South Asian people,” and “I am similar to the average British/South Asian person.” Responses were given on a 5-point Likert scale ranging from 1 (strongly disagree) to 5 (strongly agree) (Cronbach’s alpha British identity: α_White_ = .84, α_Asian_ = .77; Asian identity: Cronbach’s alpha α_White_ = .60, α_Asian_ = .83).

### Statistical analysis

Analyses were conducted using IBM SPSS Statistics 29.0 (*t*-tests, MANOVAs, Chi-square tests) and JASP Version 0.19.3 (mediation analyses). To examine correlations between all measures, Pearson’s product-moment correlations were computed separately for South Asian and White British participants. Independent samples *t*-tests were used to assess group differences in demographic characteristics, identity variables, menstrual literacy, internalized stigma of seeking help, secrecy, and ethnic microaggressions in healthcare. To test group differences in menstrual experiences and stigma outcomes, a series of multivariate analyses of variance (MANOVAs) were conducted, followed by univariate F tests. Specifically, MANOVAs were used to assess cultural group differences in: (a) menstrual distress and symptom frequency, (b) 13 symptom management strategies, (c) eight menstrual hygiene behaviors, (d) affective internalized stigma (3 subscales), and (e) 11 avoidance behaviors. Chi-square tests of independence were used to test group differences in menstrual literacy. Effect sizes were reported as partial eta squared (η^2^p) for MANOVAs and phi (φ) for chi-square tests. Mediation analyses (single mediator, two parallel mediators) were conducted to test the hypothesized indirect effects. These models were estimated using the mediation module in JASP with percentile bootstrapping (5,000 samples) to derive 95% confidence intervals for indirect effects. Cultural group was coded as White British = 0 and South Asian = 1. An alpha level of .05 was applied across all analyses. When applying a Bonferroni correction to the four *t*-tests performed and adjusting the significance threshold from α = .05 to α = .0125, the results remained unchanged.

### Researcher positionality

The research team comprised individuals from diverse backgrounds, including one White British researcher, two White European researchers, and one South Asian researcher. This diversity informed ongoing discussions and reflexive consideration of how cultural perspectives may shape the study design, analysis, and interpretation of findings. We sought to remain attentive to potential biases and to interpret results with cultural sensitivity throughout the research process.

## Results

Correlations between all measures are presented in Table S1 in the Supplementary Material.

### Sociodemographics and group identification

Sample characteristics are presented in Tables 1 and 2. White British and South Asian participants did not differ in their Subjective Socioeconomic Status. In our sample, South Asian participants were younger and reported higher levels of education than White British. Conducting the analyses controlling for age and education did not change the results. Therefore, to include the full sample (two participants did not report their education level), and in line with the preregistration, we report the analyses without controlling for age and education. White British identified more strongly with a British identity than South Asians, and South Asians reported stronger identification with an Asian identity than White British, indicating that each group identified more strongly with their home culture.

### Differences in menstrual experiences (RQ1)

#### Management of menstrual symptoms

The analysis revealed a significant multivariate effect on the 13 symptom management strategies (see Table 3), Wilks’ Lambda = .704, *F* (13, 292) = 9.42, *p* < .001, partial *η*^2^ = .296. Univariate follow-ups showed that White British reported significantly more frequent use of contraceptives and antidepressants. In contrast, South Asians reported significantly more frequent use of massage, diet, staying at home, and calling in sick at work. No significant differences were found for pain medication, exercise, heat, psychological therapy, or support from family, partner, or friends. Thus, although South Asians and White British differed in their management of menstrual symptoms, H1 was not supported.

**Table 3.** Means (standard deviations), significance tests and effect sizes for all measures of menstrual experiences as a function of cultural group.

Measures	Range	Cultural group		*F* (df1, df2)	*p*	Partial *η*2
		White British	South Asian			
**Menstrual distress**	​	​	​	*F* (1, 250)	​	​
Frequency of menstrual symptoms	​	1.04 (.36)	1.03 (.36)	0.11	.737	.000
Distress of menstrual symptoms	​	​	​	​	​	​
Distress – pain	0-3	1.48 (.65)	1.71 (.75)	6.74	.010	.026
Distress – gastrointestinal	0-3	1.27 (.72)	1.31 (.85)	0.13	.720	.001
Distress – discomfort	0-3	1.50 (.77)	1.57 (.89)	0.38	.537	.002
Distress – psychological and cognitive	0-3	1.68 (.78)	1.79 (.82)	1.15	.284	.005
Distress – physiological	0-3	1.47 (.79)	1.55 (.83)	0.55	.459	.002
**Management of menstrual symptoms**	​	​	​	*F* (1, 304)	​	​
Contraceptive	1-5	2.49 (1.68)	1.49 (1.11)	37.97	<.001	.111
Pain medication	1-5	3.53 (1.35)	3.27 (1.38)	2.67	.103	.009
Antidepressants	1-5	1.49 (1.03)	1.27 (0.76)	4.59	.033	.015
Exercise	1-5	2.80 (1.23)	2.79 (1.24)	0.00	.963	.000
Heat	1-5	3.52 (1.35)	3.59 (1.32)	0.18	.669	.001
Massage	1-5	1.96 (1.03)	2.70 (1.35)	28.84	<.001	.087
Diet	1-5	2.16 (1.20)	2.78 (1.35)	18.53	<.001	.057
Psychological therapy	1-5	1.32 (0.76)	1.50 (0.97)	3.13	.078	.010
Social support from family	1-5	2.00 (1.20)	2.14 (1.26)	0.96	.329	.003
Social support from partner	1-5	2.63 (1.46)	2.59 (1.50)	0.05	.817	.000
Social support from friends	1-5	2.08 (1.23)	2.18 (1.26)	0.54	.464	.002
Staying at home	1-5	2.92 (1.43)	3.58 (1.20)	19.22	<.001	.059
Calling in sick at work	1-5	1.71 (1.00)	2.16 (1.29)	11.70	<.001	.037
**Menstrual hygiene behaviors**	​	​	​	*F* (1, 304)	​	​
Tampons	1-7	2.70 (1.52)	1.48 (1.01)	67.87	<.001	.183
Sanitary napkins/pads	1-7	3.58 (1.46)	4.12 (1.01)	14.26	<.001	.045
Menstrual cups	1-7	1.27 (.94)	1.17 (.66)	1.27	.260	.004
Douches	1-7	1.02 (.24)	1.26 (1.08)	7.37	.007	.024
Feminine sprays	1-7	1.16 (.69)	1.38 (1.14)	4.02	.046	.013
Feminine powders	1-7	1.08 (.49)	1.12 (.59)	0.54	.462	.002
Feminine wipes	1-7	1.73 (1.35)	1.67 (1.28)	0.12	.729	.000
Other (period underwear)	1-7	1.22 (0.78)	1.16 (0.67)	0.50	.479	.002

*Note.* Sample size for all measures *N* = 306 (153 White British, 153 South Asian), apart from Menstrual Distress: *N* = 252 (126 White British, 126 South Asians).

#### Menstrual hygiene behaviors

We explored cultural group differences in eight menstrual hygiene behaviors. The multivariate effect was significant, Wilks’ Lambda = .763, *F* (8, 297) = 11.50, *p* < .001, partial η^2^ = .237, and follow-up univariate tests showed that White British reported significantly greater use of tampons, whereas South Asians reported more frequent use of sanitary napkins/pads, douches, and feminine sprays. No significant group differences were found for use of menstrual cups, feminine powders, feminine wipes, or period underwear.

#### Menstrual distress

We explored differences in frequency and distress of menstrual symptoms (see Table 4). The dependent variables were frequency of menstrual symptoms and the five clusters of distress (pain, gastrointestinal, discomfort, psychological and cognitive, and physiological). The multivariate effect was not significant, Wilks’ Lambda = .959, *F* (6, 245) = 1.76, *p* = .109, partial *η*^2^ = .041, with follow-up univariate analyses revealing a significant cultural group difference in the cluster of pain-related distress only. South Asians reported greater distress due to menstrual pain compared to White British. No significant group differences emerged for frequency of symptoms or other distress clusters.

**Table 4.** Means (standard deviations), significance tests and effect sizes for all measures of stigma of menstruation as a function of cultural group.

Measures	Range	Cultural group		*F* (df1, df2)/t (df)	*p*	Partial *η*2/*d*
		White British	South Asian			
**Affective internalized menstrual stigma**	​	​	​	*F* (1, 304)	​	​
Loathing emotions	1-5	2.34 (0.92)	2.42 (1.03)	0.51	.474	.002
Self-conscious emotions	1-5	1.60 (0.79)	1.53 (0.76)	0.60	.440	.002
Positive emotions	1-5	1.44 (0.66)	1.77 (0.82)	15.44	<.001	.048
**Internalized stigma of seeking help for menstrual problems**	1-5	1.78 (0.53)	1.87 (0.63)	*t* (304) = -1.46	.145	0.17
**Secrecy**	1-5	1.61 (0.46)	1.79 (0.64)	*t* (275.66) = -2.71	.007	0.31
**Avoidance behaviors**	1-5	​	​	*F* (1, 304)	​	​
Working outside home	1-5	1.29 (0.71)	1.80 (1.07)	23.98	<.001	.073
Washing the body	1-5	1.01 (0.11)	1.25 (0.65)	20.29	<.001	.063
Cooking/housekeeping	1-5	1.33 (0.77)	1.75 (1.01)	16.63	<.001	.052
Touching stored food	1-5	1.07 (0.36)	1.13 (0.44)	2.04	.154	.007
Exercise	1-5	1.27 (0.63)	1.73 (0.90)	26.68	<.001	.081
Talking to men about my period	1-5	1.30 (0.66)	1.68 (0.96)	16.11	<.001	.050
Talking to women about my period	1-5	1.18 (0.49)	1.24 (0.63)	1.03	.311	.003
Seeking support from male health professionals about my period	1-5	1.34 (0.71)	1.78 (1.08)	17.64	<.001	.055
Seeking support from female health professionals about my period	1-5	1.10 (0.38)	1.34 (0.77)	12.16	<.001	.038
Engaging in prayers	1-5	1.21 (0.68)	2.84 (1.72)	118.40	<.001	.280
Engaging in sexual intimacy	1-5	2.48 (1.29)	3.30 (1.52)	25.77	<.001	.078

*Note.* Sample size for all measures *N* = 306 (153 White British, 153 South Asians).

### Differences in menstrual health literacy (RQ2)

White British (*n* = 138) were more likely to attribute menstruation to a physiological cause than South Asians (*n* = 124), χ^2^ (1, N = 306) = 5.20, *p* = .023, φ = .13, supporting H2. Other attributed causes were God given (21 South Asians, 9 White British), psychological (4 White British, 4 South Asians) and “don’t know” (2 White British, 4 South Asians). Exploring the full scale, there was a significant cultural group difference in menstrual health literacy**,** with White British women scoring higher than South Asian women (see Table 2).

### Differences in menstrual stigma (RQ3)

#### Affective internalized menstrual stigma

We tested cultural group differences in three types of affective internalized stigma: loathing emotions, self-conscious emotions, and positive emotions. The multivariate test was significant (see Table 4), Wilks’ Lambda = .935, *F* (3, 302) = 7.00, *p* < .001, partial *η*^2^ = .065, with univariate analyses indicating a significant cultural difference only for positive emotions, partly supporting H3. South Asian women reported significantly more positive emotions related to menstruation than White British women. No significant differences emerged for loathing or self-conscious emotions.

#### Internalized stigma of seeking help and secrecy

No significant difference between groups was found for internalized stigma of seeking help (see Table 4). However, South Asian women reported significantly higher levels of secrecy regarding menstruation than White British women, supporting H3.

#### Avoidance behaviors

We explored cultural differences in 11 specific avoidance behaviors during menstruation. The multivariate test was significant (see Table 4), Wilks’ Lambda = .681, *F* (11, 294) = 12.497, *p* < .001, partial *η*^2^ = .319. Univariate analyses revealed that South Asian women reported significantly greater avoidance than White British women in a range of domains: working outside the home, washing the body, cooking/housekeeping, exercise, talking to men about menstruation, seeking support from male health professionals, seeking support from female health professionals, engaging in prayers, and engaging in sexual intimacy, supporting H3. No significant cultural group differences were found for avoidance of touching stored food or talking to women about menstruation.

### Differences in ethnic microaggressions in healthcare (RQ4)

South Asian and White British women differed significantly on experiences of ethnic microaggressions in healthcare**,** with South Asian women reporting more frequent experiences than White British women, supporting H4 (see Table 2).

### Mediation analyses

#### Affective internalized stigma and secrecy

Secrecy, but not affective internalized stigma, mediated the association between cultural group and avoidance behaviors (men, male health professionals, prayers, sexual intimacy as separate DVs) (see Table 5). The indirect effect through secrecy (but not affective internalized stigma) on avoidance behaviors was significant, partially supporting H5. Affective internalized stigma and secrecy did not mediate the association between cultural group and social support (family, partner, friends as separate DVs, H6a), pain medication or psychological therapy (H6b) as symptom management. None of the total indirect effects was significant (95% CI included zero). Internalized stigma of seeking help did not mediate the association between experienced ethnic microaggressions in healthcare and menstrual symptom distress (H7), the indirect effects were not significant for all five clusters (95% CI included zero).

**Table 5.** Parallel multiple outcomes mediation model: Total, direct, and indirect effects of cultural group on avoidance behaviors. Mediators: Affective internalized stigma (M1), secrecy (M2).

Path	*B*	*SE (B)*	*p*	95% CI [LL, UL]
IV-M1	0.10	0.07	.165	–
IV-M2	0.17	0.06	.008	–
**DV1 (men)**	​	​	​	​
Total effect	0.38	0.09	< .001	–
Direct effect	0.24	0.08	.002	–
M1-DV	0.09	0.08	.239	–
M2-DV	0.76	0.12	< .001	–
Indirect effect M1	0.01	0.01	–	[-0.01, 0.04]
Indirect effect M2	0.13	0.05	–	[0.03, 0.24]
Total indirect effect	0.14	0.05	–	[0.04, 0.25]
**DV2 (male health professionals)**	​	​	​	​
Total effect	0.44	0.10	< .001	–
Direct effect	0.32	0.09	< .001	–
M1-DV	0.38	0.10	< .001	–
M2-DV	0.48	0.12	< .001	–
Indirect effect M1	0.04	0.03	–	[-0.01, 0.10]
Indirect effect M2	0.08	0.03	–	[0.02, 0.18]
Total indirect effect	0.12	0.05	–	[0.03, 0.23]
**DV3 (prayers)**	​	​	​	​
Total effect	1.63	0.15	< .001	–
Direct effect	1.49	0.14	< .001	–
M1-DV	0.20	0.12	.103	–
M2-DV	0.71	0.14	< .001	–
Indirect effect M1	0.02	0.02	–	[-0.01, 0.07]
Indirect effect M2	0.12	0.05	–	[0.03, 0.24]
Total indirect effect	0.14	0.05	–	[0.04, 0.27]
**DV4 (sexual intimacy)**	​	​	​	​
Total effect	0.82	0.16	< .001	–
Direct effect	0.61	0.15	< .001	–
M1-DV	0.15	0.13	.256	–
M2-DV	1.10	0.11	< .001	–
Indirect effect M1	0.01	0.02	–	[-0.01, 0.06]
Indirect effect M2	0.19	0.07	–	[0.05, 0.34]
Total indirect effect	0.20	0.08	–	[0.05, 0.35]

*Note. B* = unstandardized coefficient, *SE* = standard error, *p* reported two-tailed, CI = percentile confidence interval, 95% CI bootstrapping (5000 resamples), *LL* = lower limit, *UL* = upper limit. Cultural group was coded as White British = 0 and South Asian = 1.

#### Menstrual literacy

We explored menstrual health literacy as the mediator of the relationship between cultural group and three stigma-related outcomes (see Table 6 and Figure 1). There were significant indirect effects through menstrual literacy on affective internalized stigma (composite score of full scale), secrecy, and avoidance behaviors (composite score of full scale). Menstrual health literacy mediated the relationship between cultural group and three stigma-related outcomes (affective internalized stigma, secrecy, and avoidance behaviors).

**Table 6.** Parallel multiple outcomes mediation model: Total, direct, and indirect effects of cultural group on affective internalized stigma (DV1), secrecy (DV2), and avoidance behaviors (DV3), mediator: Menstrual health literacy.

Path	*B*	*SE (B)*	*p*	95% CI [LL, UL]
**Total effects**	​	​	​	​
IV-DV1	.10	.07	.160	–
IV-DV2	.17	.06	.007	–
IV-DV3	.48	.05	< .001	–
**Direct effects**	​	​	​	​
Direct effect-DV1	.07	.07	.317	–
Direct effect-DV2	.13	.06	.029	–
Direct effect-DV3	.44	.05	< .001	–
**Path coefficients**	​	​	​	​
IV-M	-.26	.09	.004	–
M-DV1	-.11	.05	.023	–
M-DV2	-.16	.04	< .001	–
M-DV3	-.16	.03	< .001	–
**Indirect effects**	​	​	​	​
IV-M-DV1	.03	.02	–	[0.003, 0.06]
IV-M-DV2	.04	.02	–	[0.01, 0.09]
IV-M-DV3	.04	.02	–	[0.01, 0.08]

*Note. B* = unstandardized coefficient, *SE* = standard error, *p* reported two-tailed, CI = percentile confidence interval, 95% CI bootstrapping (5000 resamples), *LL* = lower limit, *UL* = upper limit. Cultural group was coded as White British = 0 and South Asian = 1.

**Figure 1.**
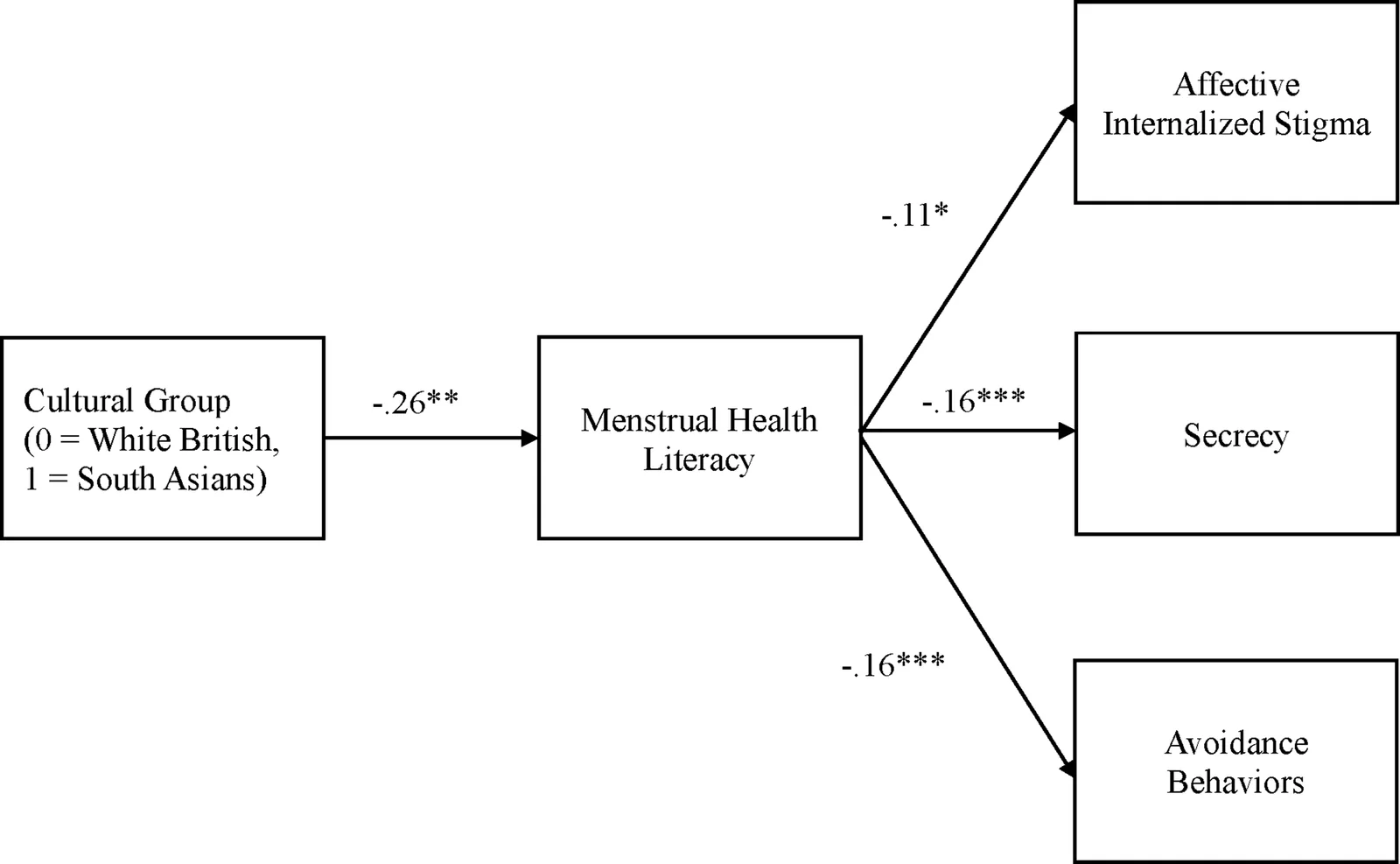
Parallel multiple outcomes mediation model: Menstrual health literacy as mediator of the relationship between cultural group and affective internalized stigma, secrecy, and avoidance behaviors. *Note*. Unstandardized coefficients are reported. ** *p* < .01, *** *p* < .001. Affective Internalized Stigma: direct effect: *B* = .07, *SE* = .07, *p* = .317; total effect: *B* = .10, *SE* = .07, *p* = .160. Secrecy: direct effect: *B* = .13, *SE* = .06, *p* = .029; total effect: *B* = .17, *SE* = .06, *p* = .007. Avoidance Behaviors: direct effect: *B* = .44, *SE* = .05, *p* < .001; total effect: *B* = .48, *SE* = .05, *p* < .001.

#### Ethnic microaggressions and secrecy

We explored secrecy and ethnic microaggressions as parallel mediators of the relationship between cultural group and internalized stigma of seeking help (see Table 7 and Figure 2). There was a significant total indirect effect. Secrecy and ethnic microaggressions mediated the relationship between cultural group and internalized stigma of seeking help.

**Table 7.** Parallel mediation model: Total, direct, and indirect effects of cultural group on internalized stigma of seeking help. Mediators: Secrecy (M1), ethnic microaggressions (M2).

Outcome variable	*B*	*SE (B)*	*p*	95% CI [LL, UL]
Total effect	.10	.07	.142	–
Direct effect	-.02	.06	.806	–
IV-M1	.17	.06	.006	–
IV-M2	.33	.06	< .001	–
M1-DV	.21	.06	.001	–
M2-DV	.23	.08	.002	–
Indirect effect M1	.04	.02	–	[0.01, 0.07]
Indirect effect M2	.08	.02	–	[0.03, 0.14]
Total indirect effect	.11	.03	< .001	–

*Note. B* = unstandardized coefficient, *SE* = standard error, *p* reported two-tailed, *CI* = confidence interval, 95% CI bootstrapping (5000 resamples), *LL* = lower limit, *UL* = upper limit. Cultural group was coded as White British = 0 and South Asian = 1.

**Figure 2.**
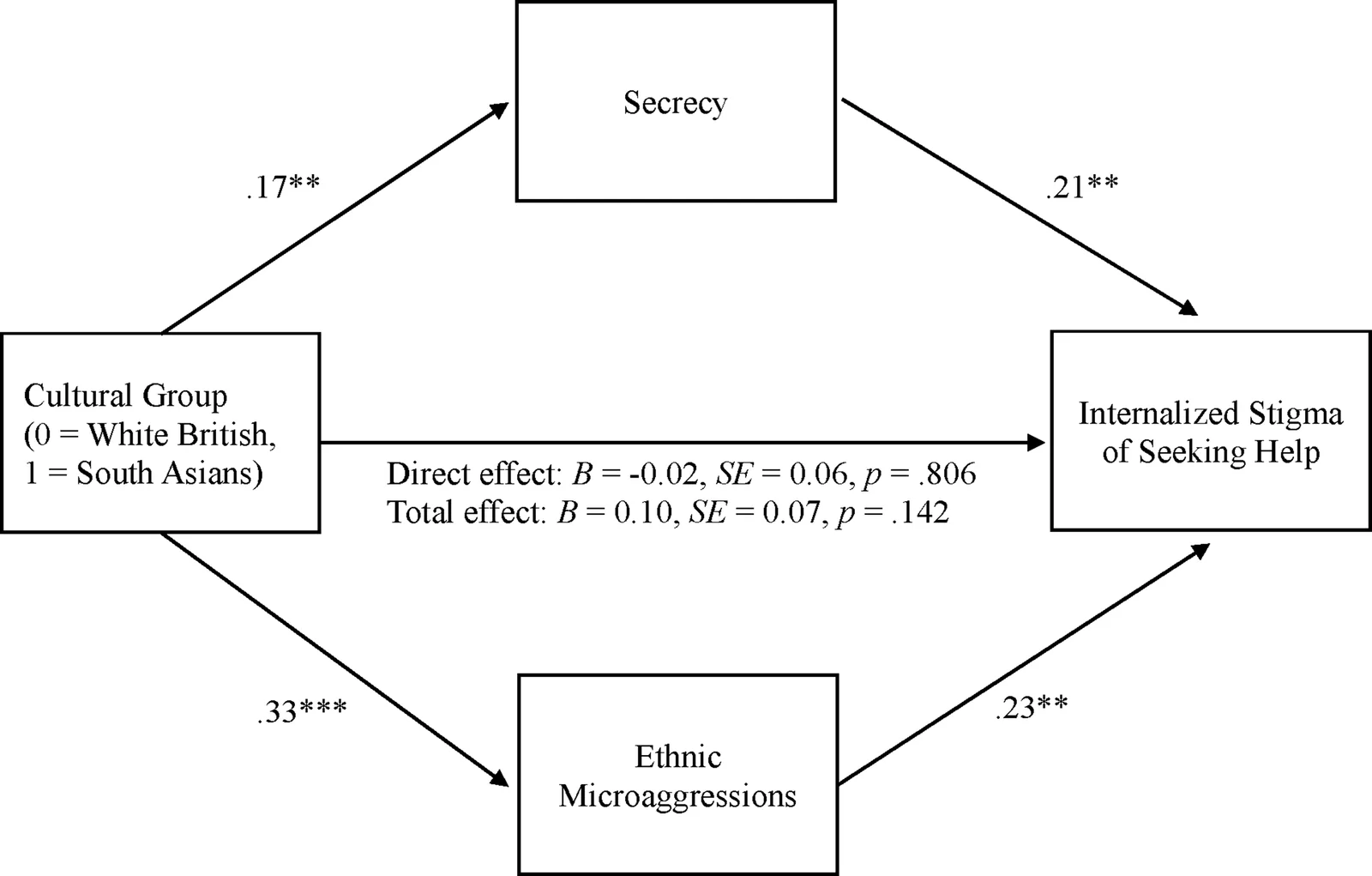
Parallel mediation model: Secrecy and ethnic microaggressions as parallel mediators of the relationship between cultural group and internalized stigma of seeking help for menstrual problems. *Note*. Unstandardized coefficients are reported. ** *p* < .01, *** *p* < .001.

## Discussion

Previous research has consistently evidenced that stigma surrounding menstruation is universal,^7^ yet suggests potential cultural differences. The present study sought to advance understanding about how menstrual health manifests across cultural groups within the same national context. By directly comparing South Asian and White British women in the UK, we investigated differences in menstrual experiences, literacy, stigma-related factors, and ethnic microaggressions in healthcare, considering group status (majority vs. minority) and associated discrimination. Our findings revealed cultural differences in how menstruation is perceived, managed, and stigmatized across groups, with important implications for health and gender equity.

First, South Asian and White British women differed in their self-reported menstrual experiences. White British women more frequently used medication (i.e., contraceptives and antidepressants), whereas South Asian women relied more on home-based (i.e., staying at home, calling in sick at work) and non-pharmacological strategies (i.e., massage and diet). This may be explained by the greater medicalization of menstruation in Western cultures.^16,17^ South Asians may also prefer traditional medicine over Western medicine.^63^ Hygiene practices differed as well, with White British more likely to use tampons and South Asians more likely to use pads, douches and feminine sprays. This mirrors previous research in which White participants were more likely to use unscented tampons and Black participants more likely to use douches and wipes, which may relate to socio-cultural differences in body taboos and appropriate practices.^22^

South Asian women reported greater distress from menstrual pain than White British women in the UK. This is in line with the mixed findings on menstrual symptoms, with some studies finding greater severity in Asian women relating to menstrual pain^12^ and general pain,^13,15^ while other studies find greater severity of premenstrual and menstrual symptoms in Caucasian women.^9–11^ Broader work shows greater pain sensitivity among minority groups.^13^

Second, South Asian women reported lower levels of menstrual health literacy, with a small-to-moderate effect size for the full scale, and a small effect size for the physiological cause item, in line with previous research which also found lower levels of menstrual literacy in Eastern compared to Western cultures.^9,25–29^ In Eastern cultures, misinformation often comes from mothers who also often have low menstrual literacy or are hesitant to openly address sensitive topics.^28,29^ Additionally, ethnic minorities living in a Western culture may still experience educational inequalities, limiting access to relevant information.^30^

Third, South Asian women showed greater menstrual stigma than White British women. They reported a stronger tendency toward secrecy and avoidance behaviors around menstruation compared to White British women (see also^28,38^). Specifically, South Asian women reported significantly more avoidance of various activities while menstruating, including talking to men about menstruation, seeking support from both male and female healthcare professionals, engaging in prayers and sexual intimacy. In South Asian cultures, menstruating women are often framed as temporarily impure and unclean, and therefore forbidden to engage in activities such as prayers or sexual intimacy.^40,41^ South Asian cultural values emphasize interdependence, emotional restraint, and the preservation of social harmony. Open discussion of taboo topics is often discouraged, with communication tending to be indirect and nonverbal. Illness and bodily matters are frequently viewed as family concerns rather than individual issues, creating pressure to protect family reputation and prevent embarrassment. These cultural dynamics help explain why South Asian women in our study demonstrated greater secrecy and avoidance when it came to menstruation.^64^ Interestingly, while stigma was generally higher among South Asian women, they also reported more positive emotional responses to menstruation, in line with the literature.^38^ The literature exploring positive menstrual experiences is currently limited. Findings on menstrual joy suggest that priming women to consider positive aspects of menstruation can change their attitudes towards menstruation, reinforcing that menstrual attitudes are influenced by cognitive framing (e.g., culture, research) rather than just physical experiences.^65,66^

Menstrual literacy, secrecy and microaggressions predicted stigma-related factors, particularly for South Asian women. Secrecy was a mediator between cultural group and avoidance behaviors, as well as affective internalized stigma, together with microaggressions as parallel mediators. Menstrual literacy emerged as a mediator between cultural group and affective internalized stigma, secrecy, and avoidance behaviors. In line with the literature, a core manifestation of menstrual stigma is the need for concealment.^33^ Stigma is perpetuated through the taboo of communication about menstruation as well as through the media, which further emphasizes ‘feeling fresh’ (scented pads), avoiding embarrassment (leaks, pads showing through clothing), and secrecy (using blue instead of red liquid).^16^ Research shows that women who think that menstruation and the cycle should be kept as secrets and feel embarrassed by them reported engaging in more behavioral change aimed at isolating themselves from the public sphere.^36^ In line with terror management theory, the female body, menstruation and its reproductive power are believed to represent a more visible reminder of the fragility and fallibility of humankind, leading to existential angst and the need for concealment.^34^ Furthermore, according to objectification theory,^35^ in societies that view women’s bodies as sexual objects, women may internalize such views and adopt a perspective on their body through the lens of a male gaze. Such self-objectification may motivate women to make extra efforts at hiding their periods.^16^ Fourth, South Asian women reported more frequent experiences of ethnic microaggressions in healthcare settings, mirroring findings that Asian Americans report being less satisfied with their doctors due to them not listening or not including them in decisions concerning their health.^67^ Our results, which point to an intersection between ethnic and menstrual stigma in predicting help seeking behaviors, are consistent with recent findings. Specifically, Webster and Iyer^68^ found significant relationships between perceived ethnic discrimination during GP consultations in the UK and medication side-effects and adherence. Together, these findings suggest that the experience of ethnic discrimination within healthcare settings is preventing people from accessing adequate care, either by discouraging medication adherence or by dampening willingness to seek help, ultimately reinforcing and maintaining widespread health inequalities.^69^

We interpret the findings primarily through the lens of cultural values and norms, guided by prior research. However, alternative explanations may also account for the observed differences, including religion, socioeconomic factors, migration background, education, and healthcare experiences. While we measured subjective socioeconomic status, migration background and healthcare experiences, future research should build on these findings to examine such additional or complementary explanations in greater detail.^70,71^

### Limitations and future research

Several limitations warrant consideration. First, the study relied on self-report measures, which are subject to social desirability bias and may not fully capture nuanced cultural experiences. The menstrual hygiene behaviors scale used in this study includes response categories with mixed temporal frames, i.e. reporting on the frequency of use in the past year, with response categories referring to monthly, weekly or daily use and “during my period”. This may introduce ambiguity in how participants interpret and report their behaviors. Although this measure was adopted from prior research^22^ to support comparability, it may not fully capture consistent or precise frequency of product use. Future studies would benefit from more standardized and clearly defined frequency-based measures. Similarly, the low internal validity of some menstrual distress clusters (i.e., pain) should be taken into consideration when interpreting the results. Future studies could investigate the menstrual distress scale reliability by cluster further, also considering the overall heterogeneity of menstrual distress items and the inherent within-person variability in symptom reporting across menstrual cycles.

Second, while we targeted two broad ethnic categories, cultural heterogeneity within these groups was not assessed. South Asian identity, for example, encompasses multiple national, religious, and linguistic backgrounds that may differentially impact menstrual health. Future research should consider examining such nuances. Third, while our sample was sufficiently powered for the planned analyses, it may not be representative of the wider UK population, particularly as participants were recruited online and may have differed in terms of digital literacy or openness to discussing menstruation.

Fourth, we did not assess gynecological conditions which may, for certain cultural groups, exacerbate menstrual symptom distress, ultimately potentially increasing avoidance behaviors (e.g., isolation, withdrawal from social activities) and reducing help‐seeking behaviors. Future studies should build on our findings and conduct a culturally sensitive analysis (e.g., ^63,64^) of how gynecological conditions might modulate the relationships found in our study, while also assessing relevant control variables (e.g., age at menstrual education, age at menarche, chronic health conditions, menstrual cycle regularity, reproductive status).

Finally, the cross-sectional design does not allow causal interpretations, especially for the mediation analyses. Future research should consider longitudinal or experimental designs to establish directionality.

### Implications

Menstrual stigma needs addressing as a determinant of health and equality. Inequalities related to menstrual health extend beyond the biological domain, affecting experiences across education, employment, and social life. By reducing ethnic and menstrual stigma and improving menstrual health, society also takes steps towards realization of the United Nations SDGs.^3^ This involves ensuring accurate and inclusive information is available in schools, families, healthcare, and the media. Embedding menstrual health education into school curricula equips young people with knowledge that challenges stigma and builds health literacy. At the same time, health systems need to provide fair and timely diagnosis and treatment of menstrual conditions, regardless of the patients’ ethnic background. Women’s health remains underrepresented in research.^1^ Expanding research on menstrual health and health discrimination will provide the evidence base necessary to inform effective interventions, guide policy development, and embed menstrual health as a central concern of global health equity.

## Conclusion

This study advances understanding of how menstrual health manifests differently across cultural groups within the same national context, taking into account majority-minority group status and the discrimination associated with minority status. Our findings highlight the importance of culturally sensitive menstrual health education, stigma-reduction initiatives, and equitable healthcare practices. Addressing both menstrual stigma and ethnic discrimination is essential for improving menstrual health outcomes and advancing global health equity.

## Supplemental Material

Supplemental Material - Period and prejudice: Cultural differences in menstrual health between South Asian and White British women Supplemental Material for Period and prejudice: Cultural differences in menstrual health between South Asian and White British women by Michèle D. Birtel, Lucia Volpi, Aisha Mirza, Laura M. König in Women's Health

## Data Availability

Preregistration, material, and data are available at https://osf.io/y6jvg.
