# Supplementary material for: Period and prejudice: Cultural differences in menstrual health between South Asian and White British women
Source: Womens Health (Lond). 2026 Sep 28;22:17455057261478339. doi: 10.1177/17455057261478339 (PMC13735779; doi:10.1177/17455057261478339)
Supplement: Supplementary file 1 [file sj-pdf-1-whe-10.1177_17455057261478339.pdf]

Supplementary Material

for

Period and Prejudice: Cultural Differences in Menstrual Health between South Asian and White British Women

Section A: Tables

Table S1

Pearson Correlations between Measures of Menstrual Health Literacy, Menstrual Stigma and Ethnic Microaggressions, for White British (Below the Diagonal, n = 153) and for South Asian (Above the Diagonal, n =153).

Measures	*1*	*2*	*3*	*4*	*5*	*6*
1. Menstrual Health Literacy	–	-.23**	-.02	-.28**	-.30**	-.13
2. Affective Internalized Menstrual Stigma	-.03	–	.26*	.13	.38**	.36**
3. Internalized Stigma of Seeking Help	-.04	.30**	–	.11	.22**	.25**
4. Secrecy	-.11	.25**	.35**	–	.47**	.02
5. Avoidance Behaviors	-.18*	.43**	.20*	.37**	–	.19*
6. Ethnic Microaggressions in Healthcare	-.07	.08	.13	-.03	.13	–

Note: *p < .05, **p < .01 (two-tailed)

Section B: Mediation Models

Figure S1

Affective Internalized Stigma and Secrecy as Proposed Mediators of the Association Between Cultural Group and Avoidance Behaviors (Men, Male Health Professionals, Prayers, Sexual Intimacy), H5

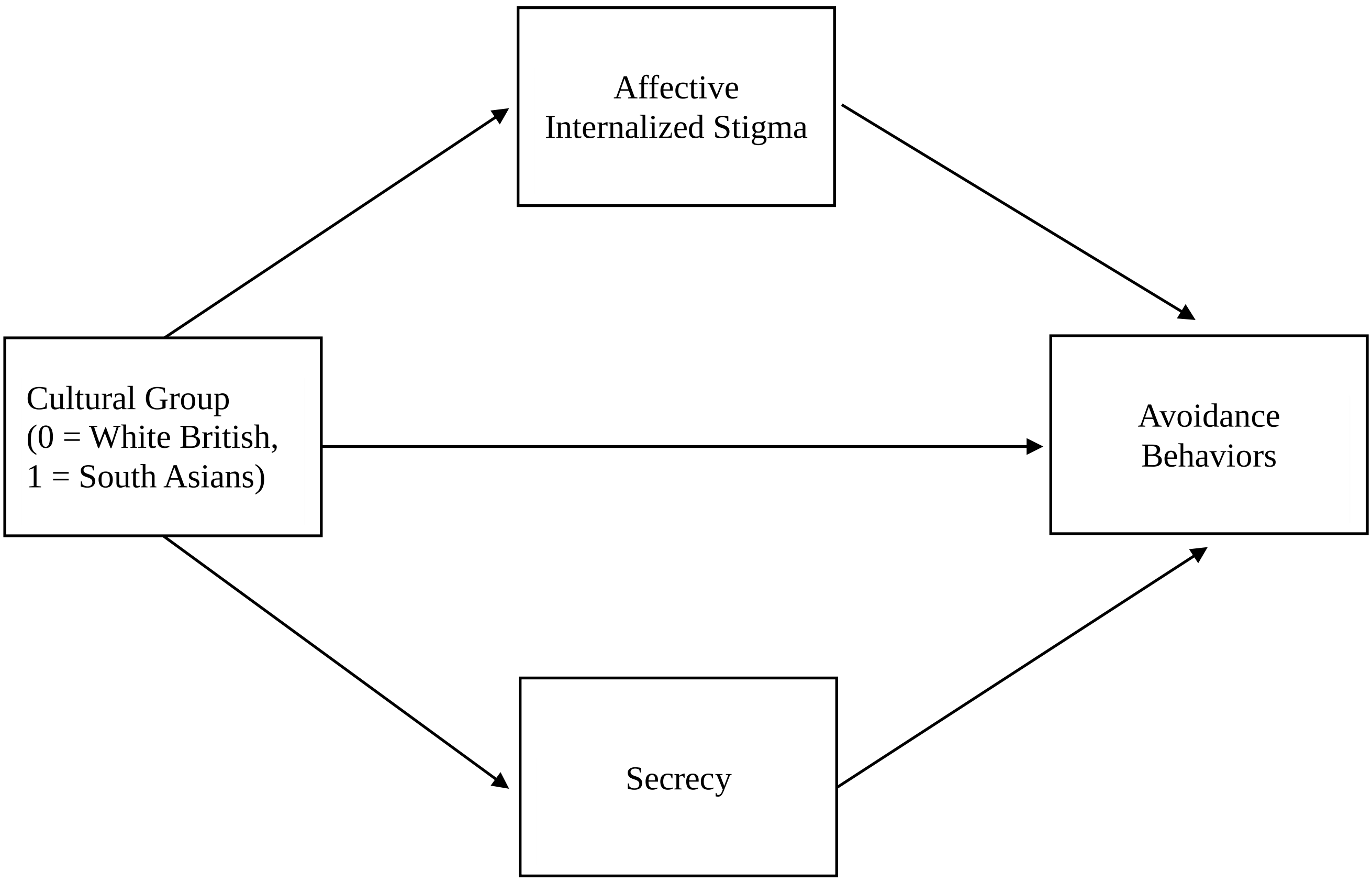

Figure S2

Affective Internalized Stigma and Secrecy as Proposed Mediators of the Association Between Cultural Group and Social Support (Family, Partner, Friends), H6a

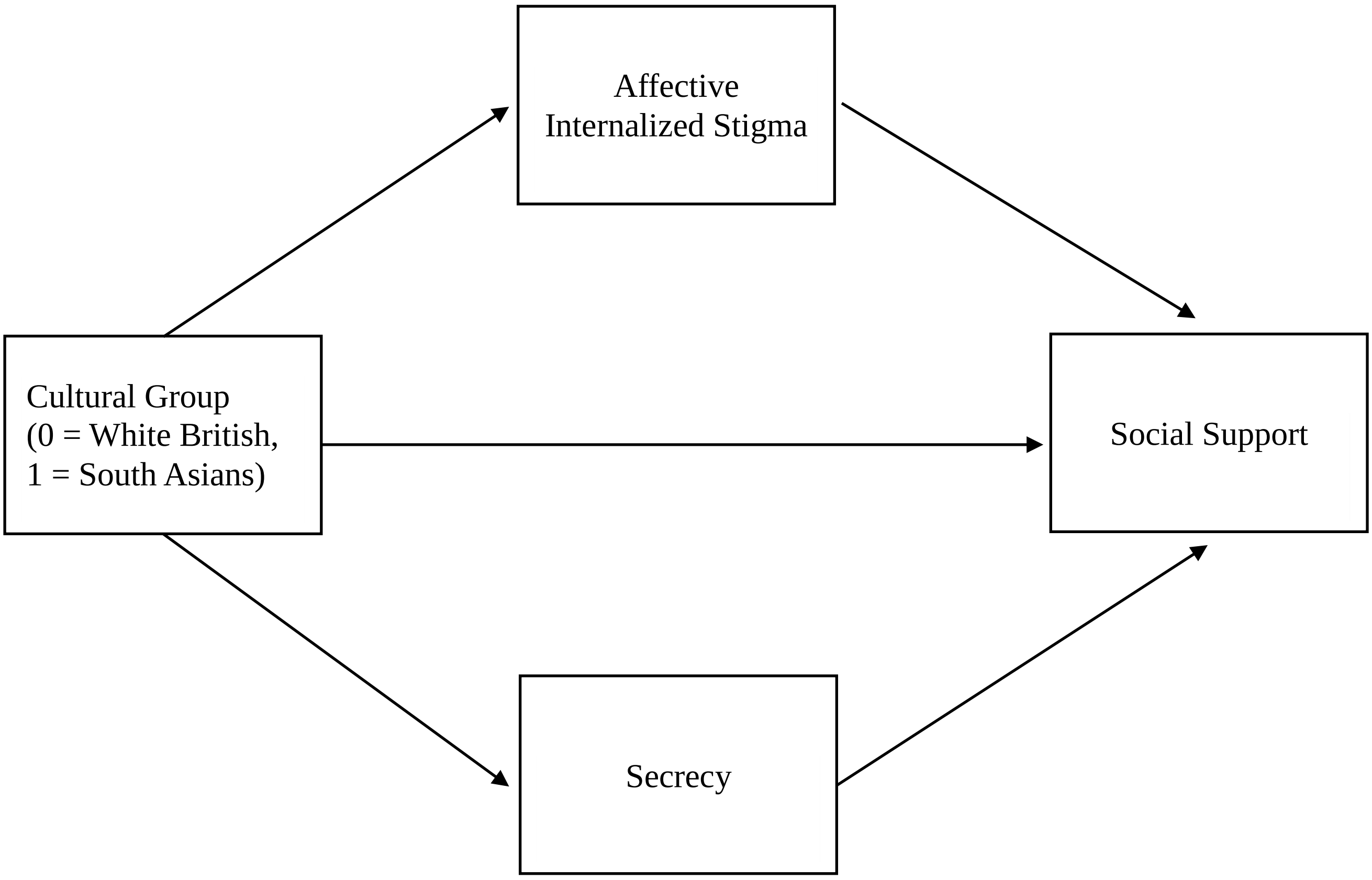

Figure S3

Affective Internalized Stigma and Secrecy as Proposed Mediators of the Association Between Cultural Group and Pain Medication as well as Psychological Therapy, H6b

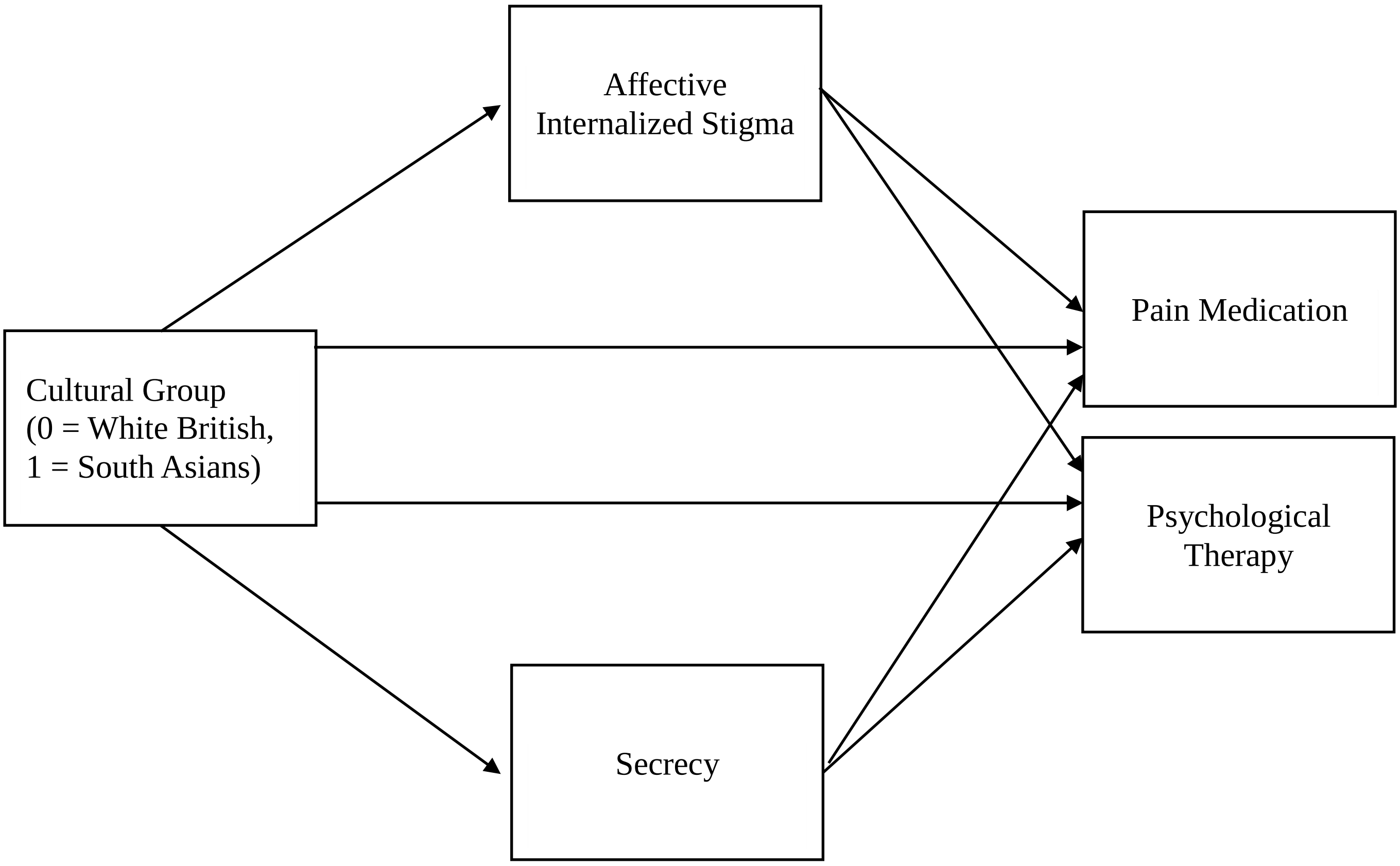

Figure S4

Internalized Stigma of Seeking Help as Mediator of the Association Between Experienced Ethnic Microaggressions in Healthcare and Menstrual Symptoms Distress, H7

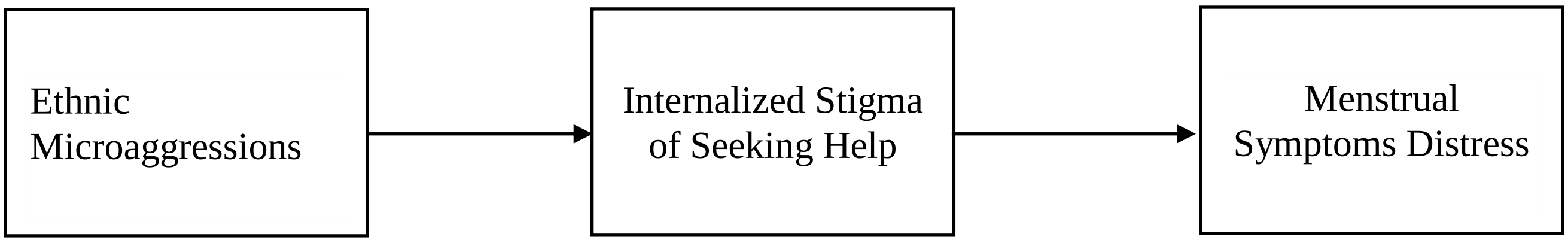

Section C: Scales

All material is also available at https://osf.io/y6jvg

Menstrual Literacy

What is the cause of menstruation?
1. Physiological
2. Psychological
3. God given
4. Result of sin
5. Due to some disease
6. Do not know

From which organ does the menstrual bleeding occur?
1. Uterus
2. Kidney
3. Bladder
4. Stomach
5. Do not know

Are there any toxins in menstrual blood?
1. Yes
2. No
3. Do not know

Are periods under the influence of hormones?
1. Yes
2. No
3. Do not know

From one menstrual period to the next, are there certain days when a woman is more likely to become pregnant?
1. Yes
2. No
3. Do not know
